# Exposure to pesticides in utero impacts the fetal immune system and response to vaccination in infancy

**DOI:** 10.1038/s41467-020-20475-8

**Published:** 2021-01-08

**Authors:** Mary Prahl, Pamela Odorizzi, David Gingrich, Mary Muhindo, Tara McIntyre, Rachel Budker, Prasanna Jagannathan, Lila Farrington, Mayimuna Nalubega, Felistas Nankya, Esther Sikyomu, Kenneth Musinguzi, Kate Naluwu, Ann Auma, Abel Kakuru, Moses R. Kamya, Grant Dorsey, Francesca Aweeka, Margaret E. Feeney

**Affiliations:** 1 Department of Pediatrics, University of California San Francisco, San Francisco, 94143 USA; 2 Department of Medicine, Zuckerberg San Francisco General Hospital, University of California San Francisco, San Francisco, 94143 USA; 3 Department of Clinical Pharmacy, University of California San Francisco, Drug Research Unit, San Francisco, CA 94143 USA; 4 Infectious Diseases Research Collaboration, Kampala, Uganda; 5 Department of Medicine, Stanford University, Stanford, 94305 USA; 6 Department of Medicine, Makerere University, Kampala, Uganda

**Keywords:** Malaria, Acute inflammation, Environmental impact, Paediatric research

## Abstract

The use of pesticides to reduce mosquito vector populations is a cornerstone of global malaria control efforts, but the biological impact of most pesticides on human populations, including pregnant women and infants, is not known. Some pesticides, including carbamates, have been shown to perturb the human immune system. We measure the systemic absorption and immunologic effects of bendiocarb, a commonly used carbamate pesticide, following household spraying in a cohort of pregnant Ugandan women and their infants. We find that bendiocarb is present at high levels in maternal, umbilical cord, and infant plasma of individuals exposed during pregnancy, indicating that it is systemically absorbed and trans-placentally transferred to the fetus. Moreover, bendiocarb exposure is associated with numerous changes in fetal immune cell homeostasis and function, including a dose-dependent decrease in regulatory CD4 T cells, increased cytokine production, and inhibition of antigen-driven proliferation. Additionally, prenatal bendiocarb exposure is associated with higher post-vaccination measles titers at one year of age, suggesting that its impact on functional immunity may persist for many months after birth. These data indicate that in utero bendiocarb exposure has multiple previously unrecognized biological effects on the fetal immune system.

## Abstract (web summary)

Control of mosquito populations using pesticides is important for malaria elimination, but effects of pesticides on humans aren’t well understood. Here, Prahl et al. show in a cohort of pregnant Ugandan women and their infants that household spraying with bendiocarb affects the fetal immune system and response to vaccination in infancy.

## Introduction

Malaria causes half a million deaths per year^1^ despite aggressive control measures that include insecticide-treated bednets and indoor residual spraying (IRS) with pesticides. IRS is a malaria reduction technique in which sprayers go door-to-door in a community and coat the interior surfaces of each home with a pesticide that persists for several months^2^. Malaria rates have been demonstrated to decline significantly after IRS^3^. Despite their widespread use, little investigation has been focused on the biological effects of pesticides, particularly on pregnant women and young infants. Prenatal exposure to some pesticides has been linked with adverse outcomes^4–6^, including a possible increase in the risk of childhood infections^7,8^. Bendiocarb, a carbamate pesticide that is one of few vector control products for IRS currently recommended by the WHO, has been shown to dramatically reduce malaria incidence^9–12^. When ingested in high doses bendiocarb causes acute anticholinesterase toxicity in humans^13^, but it is rapidly excreted in the urine, with biological effects lasting less than 24 hours^14,15^. Very limited data exist regarding the biological impact of bendiocarb exposure on humans, its systemic absorption following household spraying, its pharmacokinetics in pregnant women, or its potential effect on the developing fetus.

We examined the systemic absorption and trans-placental transfer of bendiocarb and its impact on the fetal immune system, using samples from mothers and infants enrolled in a randomized trial that compared regimens for intermittent preventive treatment of malaria during pregnancy. Among women and their infants whose houses were sprayed during pregnancy, high levels of bendiocarb were present in peripheral and umbilical cord blood, resulting in numerous alterations in fetal immune homeostasis.

## Results

### Bendiocarb is detectable in maternal plasma and is transferred trans-placentally to the fetus

**Fig. 1:**
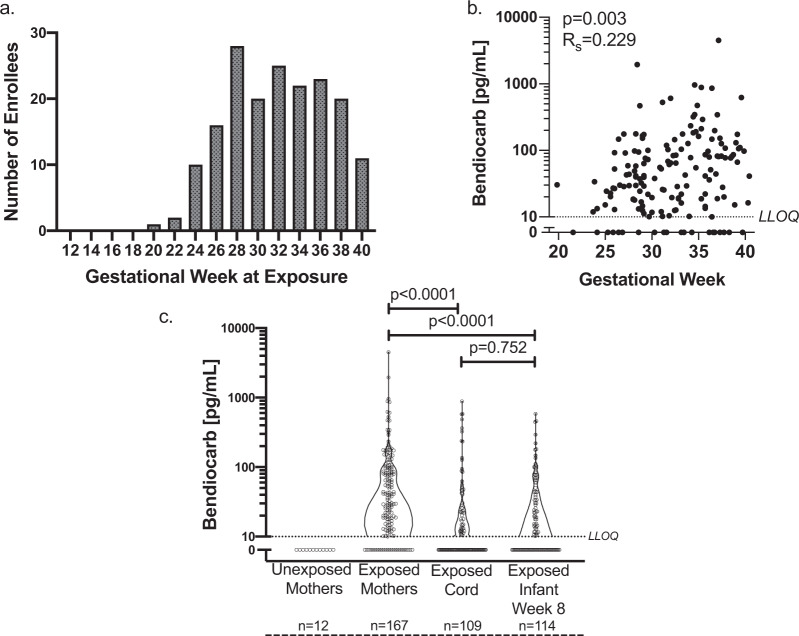
Bendiocarb is detectable at high levels in maternal plasma, is trans-placentally transferred to the fetus, and is present in infants at 8 weeks of life. **a** Timing of household bendiocarb spraying by gestational week. **b** Maternal plasma bendiocarb concentration at the time of delivery, by gestational week of exposure (*n* = 167, Spearman’s correlation). The dotted line indicates an LLOQ of 10 pg/mL. **c** Bendiocarb concentration was evaluated in available plasma from unexposed and exposed maternal plasma at delivery, exposed cord plasma, exposed infant plasma at 8 weeks of life (prior to repeat household pesticide exposure, LLOQ 10 pg/mL, Wilcoxon signed-rank test, single replicate). Bendiocarb was detected above the lower limit of quantification in 0/12 (0%) of unexposed maternal plasma at delivery, 143/167 (86%) of exposed maternal plasma at delivery, 51/109 (47%) of exposed cord plasma, and 66/114 (58%) of exposed infant plasma at 8 weeks of life. Two-sided *p* values were calculated for all test statistics.

We enrolled 300 pregnant women in the Tororo District of Uganda in a randomized trial comparing differing antimalarial regimens for the intermittent presumptive treatment of malaria during pregnancy (IPTp). Women were enrolled at 12–20 weeks’ gestation and their infants were enrolled at delivery. A total of 294 women were followed through delivery and their infants were followed through 3 years of age. Participant characteristics and study outcomes have been previously described^16^. During the study period, the Ugandan government undertook a campaign of IRS with bendiocarb to reduce malaria transmission. The homes of 177 women (60%, “exposed”) were sprayed between enrollment and delivery (20–40 weeks’ gestation; Fig. 1a), while homes of the remaining women (“unexposed”) were sprayed subsequent to delivery, except for seven subjects residing outside the targeted spraying area.

We measured bendiocarb in plasma samples using LC tandem mass spectrometry. Bendiocarb was detected in 86% of peripheral blood samples from exposed mothers at delivery, but was undetectable in 12 randomly selected unexposed mothers. Plasma concentrations in exposed women ranged from 10 to 4490 pg/mL (Fig. 1c). Levels at delivery were inversely correlated with the time elapsed since household spraying (range: 1–134 days, *p* = 0.0013, *R*_s_ = –0.248) and positively correlated with the gestational week at spraying (*p* = 0.004, *R*_s_ = 0.222, Fig. 1b). Bendiocarb was also detected in umbilical cord plasma from 47% of infants of exposed mothers (51 of 109), at lower levels ranging from 10.4 to 880 pg/mL (Fig. 1c). Bendiocarb was undetectable in four cord blood samples from unexposed mothers. Maternal and paired cord levels were strongly correlated (*p* < 0.0001, *R*_s_ = 0.521). At 8 weeks of age (prior to any additional spraying), bendiocarb was detectable in 58% of exposed infants, at levels similar to cord blood (range: 10.1–583 pg/mL; Fig. 1c). The cord blood and 8-week infant bendiocarb plasma levels were strongly correlated (*p* = 0.0005, *R*_s_ 0.399). Additionally, infant bendiocarb levels at 8 weeks of life were inversely correlated with the time elapsed since household spraying (*p* = 0.004, *R*_s_ = –0.270). Together, these data demonstrate that after household spraying, bendiocarb is systemically absorbed and trans-placentally transferred from mother to fetus.

### Bendiocarb influences fetal CD4 T cell homeostasis and increases cytokine production

**Fig. 2:**
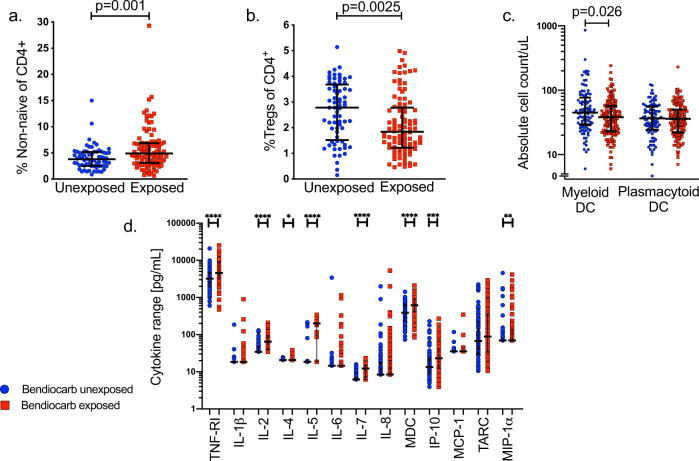
Prenatal exposure to bendiocarb impacts CD4 T cell homeostasis, cytokine levels, and dendritic cell counts in cord blood. **a** Cord blood non-naive (Supplementary Fig. 1) CD4 T cell frequency in exposed (red, *n* = 98) and unexposed (blue, *n* = 68) infants. **b** Cord blood T_reg_ frequency (CD25^high^FoxP3^+^CD127^lo^) in exposed (red, *n* = 98) and unexposed (blue, *n* = 68) infants. **c** Myeloid (Lin-2^–^HLA-DR^+^CD11c^+^CD123^-^) and plasmacytoid (Lin-2^–^HLA-DR^+^CD11c^−^CD123^+^) dendritic cell counts in whole cord blood, in exposed (red, *n* = 142) and unexposed (blue, *n* = 96) infants. **d** Cord blood plasma cytokine levels (unadjusted *p* values: TNF-RI (*p* < 0.0001), IL-2 (*p* < 0.0001), IL-4 (*p* = 0.037), IL-5 (*p* < 0.0001), IL-7 (*p* < 0.0001), MDC (*p* < 0.0001), TNF-RI (*p* < 0.0001), IP-10 (*p* = 0.0009), and MIP-1α (*p* = 0.01), ran in duplicate) in exposed (red, *n* = 114) and unexposed (blue, *n* = 73) infants. Following Bonferroni adjustment for all cytokines and chemokines measured, all associations indicated above remained significant with the exception of IL-4 and MIP-1α. All comparisons were made by Wilcoxon rank-sum test. Bars indicate median with interquartile range. Two-sided *p* values were calculated for all test statistics.

We compared immunologic parameters in the cord blood of exposed and unexposed infants. The absolute number of CD3^+^ and CD4^+^ T cells in cord blood did not differ between groups, but differences in CD4 T cell maturation, homeostasis, and cytokine production were observed. Bendiocarb-exposed infants had lower frequencies of naive CD4 cells (*p* = 0.001) and higher frequencies of CD4 cells exhibiting effector or memory differentiation (*p* = 0.001, Fig. 2a, Supplementary Fig. 1). The frequency and absolute number of FoxP3^+^ regulatory CD4 (T_reg_) cells were also lower among exposed neonates (*p* = 0.0025 and *p* = 0.01, Fig. 2b, Supplementary Fig. 2), suggesting that bendiocarb may exert a bias against regulatory differentiation, or homeostasis of these cells in utero. In support of this, cord blood T_reg_ frequencies were inversely associated with maternal bendiocarb levels at delivery (*p* = 0.011, *R*_s_ = –0.247, Table 1). Cord blood frequencies of CD4 T cells and T_reg_ cells expressing the apoptotic marker CD95 were not different between bendiocarb-exposed and unexposed infants (*p* = 0.355 and *p* = 0.113). To assess the effect of bendiocarb beyond the T cell compartment, we measured cord blood dendritic cells, chemokines, and cytokines. The cord blood of exposed infants contained fewer myeloid dendritic cells (*p* = 0.026, Fig. 2d) and higher levels of IL-2 (*p* < 0.0001), IL-4 (*p* = 0.037), IL-5 (*p* < 0.0001), IL-7 (*p* < 0.0001), MDC (*p* < 0.0001), TNF-RI (*p* < 0.0001), IP-10 (*p* = 0.0009), and MIP-1α (*p* = 0.01, Fig. 2c). Plasma levels of IL-10, TNF, IL-17A, MIG, IFN-γ, IL-12p70, and IL-23 were also analyzed but were only detectable in three or fewer samples. Cord plasma levels of IL-2, IL-5, IL-7, and TNF-RI correlated with the maternal plasma bendiocarb concentration at delivery (Table 1). Additionally, earlier gestational age at the time of exposure was associated with higher levels of IP-10 (*p* < 0.0001, *R*_s_ = –0.499), MDC (*p* = 0.005, *R*_s_ = –0.261), IL-4 (*p* = 0.003, *R*_s_ = –0.275), TARC (*p* = 0.0001, *R*_s_ = –0.353), TNF-RI (*p* = 0.034, *R*_s_ = –0.199) and lower IL-7 levels (*p* = 0.0008, *R*_s_ = 0.3104). These immunologic differences could not be readily explained by in utero pathogen exposure. Although 80% of infants in the study were prenatally exposed to malaria^16,17^, this proportion did not differ between bendiocarb-exposed and unexposed infants and there was no difference in the plasma levels of any cytokine or chemokine based on malaria exposure. Moreover, we have previously shown that malaria-exposed infants have higher frequencies of CD4 T_EM_ in cord blood^18^; thus, a reduction in malaria due to bendiocarb would be expected to bias toward lower cord blood T_EM_ frequencies. In multivariate models adjusting for prenatal malaria exposure and other potential covariates, all immune parameters with the exception of MIP-1α remained independently associated with bendiocarb exposure (Table 2).

**Table 1:** Association of immune parameters with maternal bendiocarb level at delivery.

Immune parameter	*P* value	*R*_s_
Cord blood phenotype		
T_regs_ *n* = 105	0.011	−0.247
Cord plasma cytokines		
IL–2 *n* = 121	0.046	0.182
IL–5 *n* = 121	0.015	0.220
IL–7 *n* = 121	0.007	0.246
TNF-RI *n* = 121	0.019	0.213
Cord blood-stimulated cytokines		
CD4^+^IL–2 *n* = 67	0.009	0.315
CD4^+^IL–8 *n* = 46	0.010	0.375
CD8^+^IL–2 *n* = 67	0.013	0.303
CD8^+^TNF *n* = 67	0.023	0.278
Maternal blood phenotype		
T_regs_ *n* = 64	0.088	−0.215

**Table 2:** Multivariate analysis of immune parameter by prenatal bendiocarb exposure.

	Bendiocarb exposed versus unexposed					
	Unadjusted			Adjusted^a^		
Immune parameter	Coefficient	95% [CI]	*P* value	Coefficient	95% [CI]	*P* value
Cord blood phenotype						
T_regs_	−0.54	[−0.89, −0.18]	0.004	−0.49	[−0.88, −0.09]	0.016
Myeloid DC	−22.50	[−39.82, −5.17]	0.011	−31.74	[−57.30, −5.35]	0.018
CD4^+^non-naive	1.73	[0.66, 2.80]	0.002	1.85	[0.60, 3.10]	0.004
Cord plasma cytokines						
IL–2	27.23	[16.71, 37.75]	<0.0001	31.58	[19.85, 43.36]	<0.0001
IL–4	0.76	[0.16, 1.36]	0.013	0.80	[0.12, 1.49]	0.022
IL–5	92.76	[65.77, 119.74]	<0.0001	106.13	[76.23, 136.44]	<0.0001
IL–7	3.62	[2.51, 4.72]	<0.0001	4.01	[2.80, 5.23]	<0.0001
MDC	238.52	[141.48, 335.57]	<0.0001	230.61	[119.40, 340.01]	<0.0001
TNF-RI	2342.0	[1235.28, 3448.73]	<0.0001	2318.01	[1049.77, 3596.73]	<0.0001
IP-10	9.10	[-2.56, 20.77]	0.125	13.61	[0.74, 26.58]	0.038
Cord blood-stimulated cytokines						
CD4^+^IL–2	19.88	[13.47, 26.28]	<0.0001	17.35	[10.42, 24.29]	<0.0001
CD4^+^TNF	5.59	[2.69, 8.49]	<0.0001	4.85	[1.63, 8.06]	0.003
CD4^+^IL–8	10.53	[2.89, 18.17]	0.008	13.6	[4.67, 22.52]	0.004
CD8^+^IL–2	4.01	[1.99, 6.03]	<0.0001	4.14	[1.90, 6.37]	<0.0001
Cord blood *Pf*SE proliferation						
CD4^+^CFSE^low^	−6.67	[−12.74, −0.60]	0.032	−6.42	[−13.05, 0.21]	0.058
CD8^+^CFSE^low^	−2.86	[−5.43, −0.28]	0.030	−3.71	[−6.39, −1.04]	0.007
Maternal blood phenotype	Unadjusted			Adjusted^b^		
CD4^+^Ki67^+^	−0.61	[−0.97, −0.24]	0.001	−0.58	[−0.98, −0.18]	0.005
Childhood measles vaccine response	Unadjusted			Adjusted^c^		
Measles IgG Level	3.44	[1.93, 4.95]	<0.0001	3.28	[1.38, 5.16]	0.001

Multivariate linear regression. Two-sided *p* values were calculated for all test statistics. Not adjusted for multiple comparisons.

^a^Adjusted for malaria exposure during pregnancy, maternal chemoprevention regimen, congenital CMV infection, infant sex, maternal gravidity, prematurity, and mode of delivery.

^b^Adjusted for malaria exposure during pregnancy, maternal chemoprevention regimen, maternal gravidity, prematurity, and mode of delivery.

^c^Adjusted for malaria exposure during pregnancy, episodes of malaria during childhood prior to 56 weeks, maternal and childhood chemoprevention regimen, congenital CMV infection, infant sex, maternal gravidity, prematurity, and mode of delivery.

### The T cell inflammatory response to mitogen stimulation is enhanced in bendiocarb-exposed neonates

**Fig. 3:**
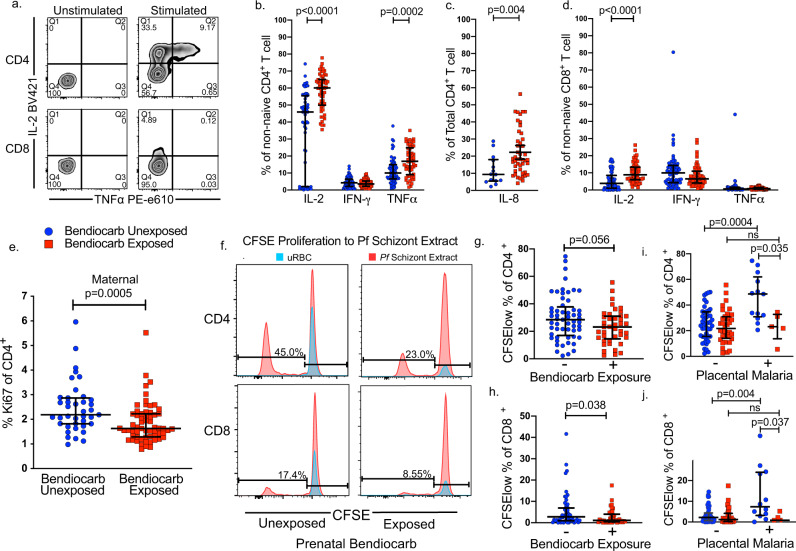
The T cell inflammatory response is enhanced but proliferation to malaria antigen stimulation is inhibited in bendiocarb-exposed maternal and cord blood. **a** A representative flow plot demonstrating the production of IL-2 and TNFα by non-naive cord blood CD4 and CD8 T cells following stimulation with PMA/io. **b** Frequencies of cytokine-producing cord blood non-naive CD4 T cells for IL-2, IFN-γ, and TNFα following stimulation with PMA/io in exposed (red, *n* = 57) and unexposed (blue, *n* = 63) infants. **c** Frequencies of cytokine-producing cord blood total CD4 T cells for IL-8 following stimulation with PMA/io in exposed (red, *n* = 46) and unexposed (blue, *n* = 14) infants. **d** Frequencies of cytokine-producing cord blood non-naive CD8 T cells following stimulation with PMA/io in exposed (red, *n* = 57) and unexposed (blue, *n* = 63) infants **e** Frequencies of maternal CD4 T cells expressing the proliferation marker Ki67 in exposed (red, *n* = 61, red) and unexposed (blue, *n* = 39) mothers. **f** Cord blood T cells were stimulated with P. *falciparum* schizont extract (red) or uninfected red blood cells (uRBCs, blue) and proliferation was measured by dilution of CFSE in CD4 and CD8 T cells. A representative plot of CD4 and CD8 T cell proliferation in a bendiocarb-unexposed (left) and exposed (right) infant. Frequency represents CFSE dilution to stimulus prior to background subtraction. **g**, **h** CD4 and CD8 T cell proliferation in response to PfSE was lower in infants prenatally exposed to bendiocarb in exposed (red, *n* = 41) and unexposed (blue, *n* = 57) infants. **i**, **j** When stratified by active placental malaria, CD4 and CD8 T cell proliferation to PfSE was robust in bendiocarb-unexposed infants, but ablated in bendiocarb-exposed infants (*n* = 98). All comparisons use Wilcoxon rank-sum test. Bars indicate median with interquartile range. Two-sided *p* values were calculated for all test statistics.

To assess the impact of bendiocarb on T cell function, we measured cord blood responses to mitogen stimulation in vitro. Following stimulation with PMA/ionomycin, bendiocarb-exposed infants had higher percentages of non-naïve CD4 cells producing IL-2 (*p* < 0.0001) and TNFα (*p* = 0.0002, Fig. 3b). Production of IL-8, a cytokine that is often produced by naive cells in the neonate, was increased among total CD4 T cells (*p* = 0.004, Fig. 3c). The percentage of non-naive CD8 cells producing IL-2 (<0.0001) was similarly increased (Fig. 3d). These differences were independent of malaria exposure (Table 2) and remained statistically significant if the cohort was restricted to the 80% of infants who were exposed in utero to malaria. Enhancement of T cell mitogen responses was directly correlated with maternal bendiocarb plasma levels (Table 1), suggesting a dose–response relationship.

### Bendiocarb exposure inhibits proliferation of fetal and maternal T cells

We next examined T cell proliferation by bendiocarb exposure. At delivery, bendiocarb-exposed mothers had lower percentages of Ki67^+^ CD4 cells (*p* = 0.0005, Fig. 3e), indicating reduced proliferation in vivo. To determine whether bendiocarb exposure inhibits T cell proliferation in response to a recently encountered antigen, we measured the infant response to *P. falciparum* by CFSE dilution. We have previously shown that infants with placental malaria exhibit robust CD4 and CD8 cell proliferation to *P. falciparum* schizont extract (PfSE)^18^. Consistent with this, CD4 and CD8 cell proliferation in response to PfSE was observed in bendiocarb-unexposed infants who were born to mothers with active placental malaria (*p* = 0.0004 and *p* = 0.004; Fig. 3i, j): however, this response was ablated in infants prenatally exposed to bendiocarb (Fig. 3i, j). Together, these data suggest that antigen-driven proliferation may be inhibited by bendiocarb.

### Prenatal bendiocarb exposure is associated with enhanced response to measles vaccination in infancy

**Fig. 4:**
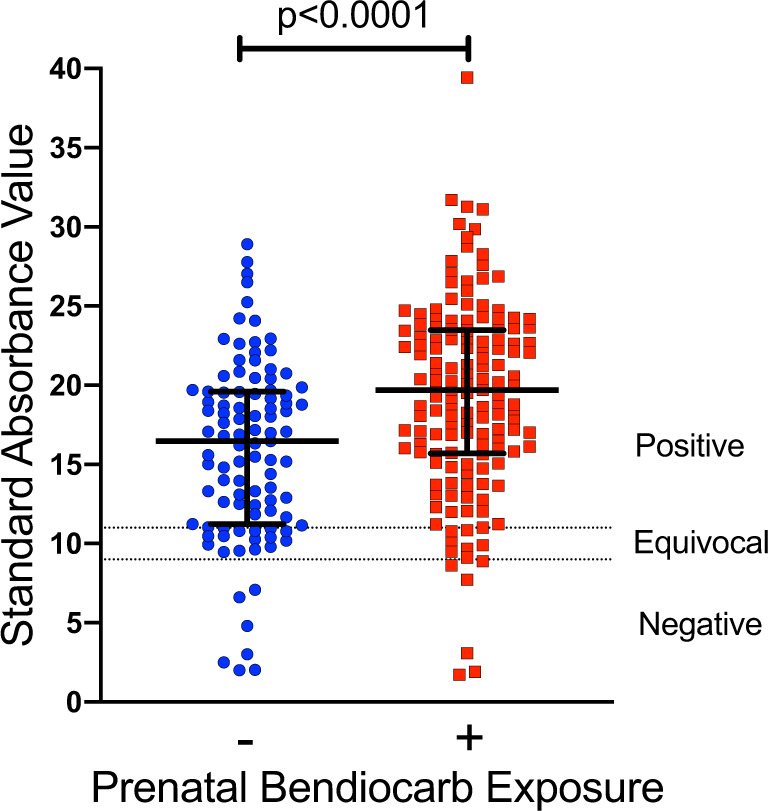
Prenatal bendiocarb exposure is associated with higher post-vaccination levels of measles-specific IgG. Measles-specific IgG levels at 1 year of age among infants prenatally exposed to bendiocarb (exposed, red, *n* = 149 and unexposed, blue, *n* = 99, *p* < 0.0001, ran in duplicate). Dotted lines indicate control thresholds (Wilcoxon rank-sum test, bars indicate median with interquartile range. Two-sided *p* values were calculated for all test statistics.

Finally, to determine the impact of prenatal bendiocarb exposure on antigen-specific immune function, we evaluated the response to postnatal measles vaccination. All infants received a single dose of measles vaccine at 9 months of age and we measured anti-measles IgG in plasma obtained at 1 year of age. Overall, 85.9% were measles IgG-positive, 8.9% were equivocal, and 5.2% were IgG-negative. Both the level of anti-measles IgG (*p* < 0.0001, Fig. 4) and the proportion of infants who were measles IgG-positive following vaccination (91.3% vs. 77.8%, *p* = 0.008) were significantly higher among bendiocarb-exposed infants. Measles IgG positivity did not differ by in utero malaria exposure, sex, gravidity, or the number of malaria episodes during the first year of life, although we cannot exclude a role for unmeasured confounders including postnatal bendiocarb exposure during subsequent rounds of IRS. Moreover, the association between post-vaccination measles titers and prenatal bendiocarb exposure remained significant following adjustment for all covariates (Table 2). These data suggest the possibility that prenatal bendiocarb may have a durable impact on functional immunity in the infant.

## Discussion

Pesticides are important tools for malaria control but may have unanticipated effects on human health. We found that bendiocarb, a carbamate pesticide in common use worldwide, is absorbed in pregnant women, transferred to the fetus, and remains detectable in infancy. Exposure to bendiocarb in utero had clear biological effects on the fetal immune system, independent of its impact on malaria vector control. These included dose-dependent alterations in immune cell homeostasis and function, encompassing both lymphoid and myeloid-derived lineages, including decreased FoxP3^+^ regulatory CD4 cells, increased cord plasma cytokines and chemokines, increased T cell production of inflammatory cytokines, and decreased T cell proliferation. Together, these changes suggest a shift in fetal T cell homeostasis toward an inflammatory response, and away from regulatory differentiation that is believed to be critical for the maintenance of maternofetal tolerance^19,20^.

The mechanism by which bendiocarb impacts the immune system is not known. Some carbamate pesticides are ligands of the aryl hydrocarbon receptor^21^, which regulates both innate and adaptive immunity, and are known to alter T cell differentiation, including T_regs_ and Th17 cells^22–25^. Dithiocarbamate pesticides, which are carbamate structural analogs, have been shown to inhibit activation of NFkB-mediated signaling in human CD4 cells, leading to reduced IL-2 production and decreased expression of CD25 (IL-2-Rα)^26–28^. Alternatively, it is possible that bendiocarb may upregulate pro-apoptotic pathways, as carbamate pesticides have been previously shown to increase cellular apoptosis of human T lymphocytes in vitro^29^, although we did not observe increased expression of the apoptotic marker CD95 in bendiocarb exposed individuals. Lastly, bendiocarb has been shown to inhibit in vitro proliferation in mammalian cell lines^15^. Further studies are needed to define the cellular and molecular targets of bendiocarb.

It is critical that future studies define the pharmacokinetics of bendiocarb in pregnant women and infants. We observed high levels in women at delivery, often several months after spraying of their homes. Because bendiocarb is believed to undergo rapid renal elimination^13,14^, this likely reflects ongoing environmental exposure to pesticide residue in the household. Moreover, many infants had detectable bendiocarb at 8 weeks of life, prior to repeat spraying, suggesting either delayed renal clearance or postnatal exposure through environmental contact in the home, or through breast milk. (All infants were exclusively breastfed, but breast milk samples were not collected.) Prior cross-sectional studies in urban US cohorts have reported trace levels of bendiocarb in pregnant women and paired cord blood, presumed to be due to unmeasured household pesticide use^30,31^. However, the maximum bendiocarb levels observed in earlier studies were more than 100-fold lower than in the current study, and its biological impact on the fetus was not investigated.

The clinical implications of in utero bendiocarb exposure cannot be easily predicted. By skewing fetal immune homeostasis toward enhanced inflammation and diminished immunoregulation, bendiocarb could potentially result in adverse pregnancy outcomes. Because all participants in this study were exposed during the second half of pregnancy, we were unable to assess the impact of exposure to bendiocarb early in pregnancy, but teratogenicity has not been reported in animal studies. The finding of higher of post-vaccination measles titers among infants exposed to bendiocarb in utero suggests that the impact of prenatal exposure persists through the first year of life, influencing the immune response to vaccination with a novel antigen at 9 months of age. This somewhat paradoxical observation of enhanced vaccine responsiveness indicates that the immune changes induced by bendiocarb may not be entirely detrimental, although we were unable to demonstrate an impact on clinical outcomes. We did not observe any impact of prenatal bendiocarb exposure on malaria incidence during infancy, although such an effect may have been obscured by postnatal IRS and antimalarial chemoprevention.

Our findings strongly indicate that environmental pesticide use can result in systemic absorption and transplacental exposure of the fetus, and may have important biological consequences for fetal development and infant health. The risks of pesticide use as a vector control tool must be balanced against the important goal of preventing childhood and pregnancy-associated malaria, which causes tremendous morbidity worldwide. There is a clear need for further research on the short- and long-term physiological effects of pesticide exposure, including its impact on fetal development and immunity, to guide risk–benefit assessments of the use of IRS.

## Methods

### Cohort characteristics

Institutional review boards of the Uganda National Council of Science and Technology (UNCST), Makerere University, and the University of California San Francisco (UCSF) approved the study. Informed consent was obtained from all study participants. Three hundred HIV-negative pregnant women were enrolled between 12–20 weeks' gestation and randomized to receive either dihydroartemisinin-piperaquine (DP) or sulfadoxine-pyrimethamine (SP) for intermittent preventive treatment of malaria (ClinicalTrials.gov number, NCT02163447)^16^. Two hundred ninety-four children were born between October 2014 and May 2015. Clinical characteristics of the cohort by prenatal bendiocarb exposure are listed in Supplementary Table 1. After birth, children were randomized to receive dihydroartemisinin-piperaquine monthly or every 12 weeks from 8 weeks until 24 months of age and were followed clinically through 3 years of age. Cord blood plasma and cells were cryopreserved, along with longitudinal peripheral blood mononuclear cell (PBMC) and plasma samples from both mothers and infants. Homes in the Tororo District were sprayed with bendiocarb between December 2014 and February 2015, and the precise date of spraying was recorded for each dwelling. Additional rounds of indoor residual spraying (in the study district during the trial) were done in June–July 2015 (when infants were between 4 and 40 weeks' of age), and then again in November–December 2015, June–July 2016, and July–August 2017.

### Evaluation for malaria

Tororo is a holoendemic region in which malaria occurs year-round. Mothers were evaluated for malaria parasitemia every 4 weeks by loop-mediated isothermal amplification (LAMP). Febrile episodes were evaluated by blood microscopy and, if positive, treated for malaria per local guidelines. Placentas were evaluated by microscopy, LAMP, and histopathology^16^. Infants were categorized as malaria-exposed in utero if their mothers had any positive testing for malaria during pregnancy or at delivery, or evidence of placental malaria by histopathology. Active placental malaria was defined by a positive LAMP assay of placental blood.

### Mass spectrometry

A sensitive LC/MS/MS method was developed to measure bendiocarb levels in human plasma. Bendiocarb was extracted from plasma samples using solid-phase extraction cartridges in a 96-well format plate. Extracted samples were then loaded onto a Waters Ultra Performance Liquid Chromatography (UPLC) instrument, and resolved using an Agilent ZORBAX Rapid Resolution High Definition (RRHD) C8 column with a gradient of ammonium formate buffer and acetonitrile mobile phases. A Sciex 6500 triple quadrupole mass spectrometer in positive mode with electrospray ionization was used for detection. The method uses a linear regression standard curve with a range from 10 to 1000 pg/mL to estimate bendiocarb sample concentrations with a deuterated bendiocarb standard as the internal control.

### Flow cytometry T cell phenotyping

CBMCs and maternal PBMCs were thawed, aliquoted at 1 × 10^6^ cells, cells were washed, stained, fixed, and permeabilized, as per the manufacturer’s instructions (FoxP3 Fix/Perm kit; eBiosciences) using standard protocols using the following antibodies: APC/Cy7 CD3 (clone OKT3), PerCP CD4 (clone RPA-T4), Brilliant Violet 421 CD25 (clone BC96), Brilliant Violet 650 CD127 (clone A019D5), Brilliant Violet 605 CD45RO (clone UCHL1), FITC CCR7 (clone G043H7), Brilliant Violet 510 CD8 (clone SK1), Brilliant Violet 510 CD14 (clone M5E2), Brilliant Violet 510 CD19 (clone HIB19, BioLegend), FITC Ki67 (clone Ki67, BD Pharmingen), PE FoxP3 (clone PCH101, eBioscience), and LIVE/DEAD aqua amine (Invitrogen). Flow cytometry data were collected on an LSR II four-laser flow cytometer (BD) with FACSDiva software. Color compensation was performed using compensation beads. Fluorescence-minus-one samples were used to define negative and positive populations. Cellular profiles were gated on live, single-cell, dump negative (CD14, CD19, CD8), CD3 + lymphocytes.

### Dendritic and CD4 T absolute cell counts

Dendritic cells and CD4 T cells were enumerated from 50 μL of whole cord blood stained with antibodies in BD TruCount™ tubes or with 20 μL of CountBright™ counting beads (ThermoFisher Scientific). Cells were incubated for 20 min and 900 μL of BD FACS lysis solution was added for 15 min. CD4 T cell staining was performed on 152 cord blood samples using PerCP CD3 (Clone SK7), APC CD4 (Clone RPA-T4, BD Pharmingen) antibodies. Dendritic cell staining was performed on 238 cord blood samples using FITC Lin-2 (clone MφP9/SJ25C1/SK7/L27/NCAM16.2, anti-CD3, CD14, CD19, CD20, CD56), PE CD123 (clone 9F5), PerCP HLA-DR (clone L243), and APC CD11c (clone S-HCL-3, BD Pharmingen). Cells were immediately analyzed on an Accuri A6 cytometer. Dendritic cells were defined as Lin-2^−^HLA-DR^+^, myeloid dendritic cells were defined as Lin-2^−^HLA- DR^+^CD11c^+^CD123^−^, and plasmacytoid cells were defined as Lin-2^−^HLA-DR^+^CD11c^−^CD123^+^. To normalize the frequency of analyzed CD4 subsets (T_reg_ cells) from cryopreserved CBMCs to absolute rates of CD4 per microliter of fresh whole cord blood, absolute CD4 subset counts were calculated (CD4 subset frequency * absolute CD4 count per microliter of whole cord blood).

### Cytokine and chemokine measurement

The concentrations of 20 cytokines and chemokines were measured in plasma samples using Luminex technology (Luminex Corporation, Austin, TX, USA). Fourteen cytokines (TNF, TNF-RI, IFN-γ, IL-1β, IL-2, IL-4, IL-5, IL-6, IL-7, IL-8, IL-10, IL-12p70, IL-17, IL-23) and six chemokines (MIG, MDC, MIP-1α, MCP-1, IP-10, TARC) were analyzed using a custom R&D Magnetic Luminex Screening Assay Human Premixed Multi-Analyte Kit (Catalog Number LXSAHM). Plasma was diluted 1:2 as per the manufacturer’s recommendations. All samples were analyzed in duplicate. Protocols were performed as indicated by the vendor. The plate was read on a Luminex MAGPIX CCD Imager and data were analyzed using xPONENT software (v4.2). Minimums of 50 beads per region were analyzed. A curve fit was applied to each standard curve according to the manufacturer’s instructions and sample concentrations were interpolated from the standard curves. MFIs were background subtracted. The variability between duplicates was minimal, with an average coefficient of variance of 5.2%. The limit of detection for each analyte was as follows: TNF: 14.83 pg/ml, TNF-R1: 220.58 pg/ml, IFN-γ: 7.55 pg/ml, IL-1B: 17.75 pg/ml, IL-2: 30.62 pg/ml, IL-4: 18.26, IL-5: 17.57 pg/ml, IL-6: 14.03 pg/ml, IL-7: 5.84, IL-8: 8.13 pg/ml, IL-10: 13.64 pg/ml, IL-12p70: 278.45 pg/ml, IL-17a: 21.42 pg/ml, IL-23: 239.36, MIG: 396.06 pg/ml, MDC: 59.01 pg/ml, MIP-1α: 60.39 pg/ml, MCP-1: 32.70 pg/ml, IP-10: 3.78 pg/ml, TARC: 9.06 pg/ml. Samples below the limit of quantification were given a value at the limit of detection. A number of cord plasma cytokines were analyzed but found to only have three or fewer samples with cytokine levels above the lower limit of detection: IL-10 (*n* = 2), TNF (*n* = 2), IL-17A (*n* = 1), MIG (*n* = 1), IFN-γ (*n* = 0), IL-12p70 (*n* = 1), IL-23 (*n* = 3).

### T cell response to mitogen stimulation

Thawed CBMCs (1×10^6^ cells/condition) were stimulated with phorbol 12-myristate 13-acetate (0.1 μg/mL) and ionomycin (1.0 μg/mL) or media alone (R10; 10% fetal bovine serum) for 5 h at 37 °C. Brefeldin A and monensin (BD Pharmingen) were added after 1 h of incubation at a final concentration of 10 mg/mL. After 5 h, cells were washed, fixed and permeabilized, as per the manufacturer’s instructions (FoxP3 Fix/Perm kit; eBiosciences), then stained with PE-Cy5.5 CD3 (Clone SK7), PE-e610 TNFα (clone MAB11, eBioscience), BV570 CD4 (Clone RPA-T4), BV711 CD8 (clone RPA-T8), BV650 CD45RA (clone HI100), BV605 CD45RO (clone UCHL1), BV510 γδTCR (clone B1), BV510 CD14 (Clone M5E2), BV510 CD19 (Clone HIB19), PE-Cy7 IFN-γ (Clone 4 S.B.3), BV421 IL-2 (clone MQ1-17H12), AF488 IL-8 (clone MQ1-17H12, Biolegend), and LIVE/DEAD aqua amine (Invitrogen). Data were collected on an LSR II (BD) and analyzed with FlowJo software (Treestar). Cord blood CD4 and CD8 T cell cytokine responses to mitogen stimulation were gated on non-naive effector populations by expression of CCR7 and CD45RO (as in Supplementary Fig. 1) for all except IL-8, which was gated on total CD4 T cells.

### *P. falciparum* schizont extract preparation

*P. falciparum* schizont extract (*Pf*SE) was prepared by three freeze–thaw cycles of infected red blood cells (iRBCs) in liquid nitrogen and a 37 °C water bath. PfSE was then resuspended in R10 media and stored at –20 °C. Uninfected red blood cells (uRBCs) were used as controls.

### CFSE proliferation assay

The proliferative response to *P. falciparum* schizont extract (*Pf*SE) was assessed by CFSE dilution. For subjects with sufficient CBMCs (*n* = 98), thawed CBMCs were rested for 1 h at 37 °C, washed in 10% Human AB (HAB) medium, and 3–4 × 10^6^ cells were labeled with 1 μM CFSE (Molecular Probes) for 7 min. CFSE-labeled CBMCs were plated in 96-deep-well culture plates (Nunc) (1 × 10^6^ cells/condition) and incubated with *Pf*SE, uninfected red blood cells (uRBCs), or media alone. After 6 days, cells were washed twice in culture medium, then stained with PE-Cy5.5 CD3 (Clone SK7) eBioscience, BV570 CD4 (Clone RPA-T4), BV711 CD8 (clone RPA-T8), BV510 CD14 (Clone M5E2), BV510 CD19 (Clone HIB19) Biolegend, LIVE/DEAD aqua amine (Invitrogen) and counted.

### Measles IgG measurement

The IgG response to measles virus was assessed using commercially available anti-measles virus IgG Human ELISA kit (Abcam, ab108750). Cryopreserved plasma obtained at 56 weeks of life was thawed and analyzed in duplicate as per the manufacturer’s guidelines using paired capture and detection antibodies. Briefly, 1:100 μL sample-to-sample diluent was incubated on a pre-coated measles virus ELISA plate, which included measles virus IgG positive control, cut-off control, and negative control. Absorbance was read at 450 nm on a SpectraMax M2 microplate ELISA reader. All absorbance values were mean background subtracted to standard units based on the cut-off control. Samples were considered positive if the mean absorbance was more than 10% over the cut-off value, considered negative if the mean absorbance value was lower than 10% below the cut-off value, and considered equivocal if the mean absorbance value was less than 10% above or below the cut-off value.

### Statistical analysis

Statistical analyses were performed using PRISM 8.0 (GraphPad) and STATA 14 (StataCorp).

The Wilcoxon rank-sum test was used for two-group comparisons of continuous variables. Paired measures were compared using the Wilcoxon sign-rank test for non-parametric variables. Associations between continuous variables were assessed using Spearman’s rank correlation (R_s_). Multivariate linear regression models for infant parameters controlled for prenatal malaria exposure, maternal chemoprevention regimen, congenital CMV infection, infant sex, maternal gravidity, prematurity, and mode of delivery (degrees of freedom, 8). Multivariate linear regression models for maternal parameters controlled for prenatal malaria exposure, maternal chemoprevention regimen, maternal gravidity, prematurity, and mode of delivery (degrees of freedom, 6). Multivariate linear regression models for vaccine responses at 56 weeks of life controlled for prenatal malaria exposure, maternal and child chemoprevention regimen, congenital CMV infection, infant sex, maternal gravidity, prematurity, mode of delivery, and episodes of malaria prior to 56 weeks of age (degrees of freedom, 9). No statistical evidence for interaction in a linear regression model was found; thus, we considered bendiocarb exposure and malaria exposure independent in our models. Additionally, we completed a sensitivity analysis to evaluate the effect of different definitions of in utero malaria exposure (placental histopathology, placental LAMP, and/or evidence of maternal infection during pregnancy) and found comparable results to our models. The incidence of malaria was compared between prenatal bendiocarb exposure groups using negative binomial regression. Associations between measles IgG positivity and bendiocarb exposure were compared using chi-squared testing. Two-sided *p* values were calculated for all test statistics, and *p* < 0.05 was considered significant.

### Reporting summary

Further information on research design is available in the Nature Research Reporting Summary linked to this article.

## Supplementary information

Supplementary Information

Peer Review File

Reporting Summary

## Data Availability

The datasets generated and/or analyzed during the current study are available from the corresponding author on reasonable request. Source data are provided with this paper.

## Source data

Source Data
