# Supplementary material for: Exposure to pesticides in utero impacts the fetal immune system and response to vaccination in infancy
Source: Nat Commun. 2021 Jan 8;12:132. doi: 10.1038/s41467-020-20475-8 (PMC7794579; doi:10.1038/s41467-020-20475-8)
Supplement: Supplementary file 1 — Supplementary Information [file 41467_2020_20475_MOESM1_ESM.pdf]

Supplementary Table 1. Clinical characteristics by prenatal bendiocarb exposure

	No Prenatal Bendiocarb Exposure n=117 (n, %)	Prenatal Bendiocarb Exposure n=177 (n, %)	p-value
**Maternal Chemoprevention arm**			
3-Dose SP	35 (29.9%)	66 (37.3%)	
3-Dose DP	42 (35.9%)	51 (28.1%)	
Monthly DP	40 (34.2%)	60 (33.9%)	p=0.327
**Infant Sex**			
Male	69 (59.0%)	79 (44.6%)	p=0.016
**Maternal Gravidity**			
Primagravida	56 (47.9%)	46 (26.0%)	
Multigravida	61 (52.1%)	131 (74.0%)	p<0.001
**Prematurity**			
<37 weeks gestational age	21 (18.0%)	8 (4.5%)	p<0.001
**Mode of Delivery**			
Caesarean	4 (3.4%)	18 (10.2%)	p=0.031
**Maternal Malaria during Pregnancy**			
Positive for clinical malaria and/or blood parasitemia and/or placental malaria	92 (78.6%)	144 (81.4%)	p=0.566
**Congenital CMV Infection**			
PCR-positive at birth[A]	3 (3.7%)	4 (3.9%)	p=0.960
**Childhood Clinical Malaria**	Incidence (Number of malaria episodes)		
Prior to 56 weeks of life	0.094 (10)	0.093 (15)	p=0.995

Listed analyses χ^2 with the exception of childhood clinical malaria which was compared by negative binomial regression. Two-sided p values were calculated for all test statistics. Not adjusted for multiple comparisons.

Abbreviations: SP, sulfadoxine-pymethamine; DP, dihydroartemisinin-piperaquine

[A]Samples screened for congenital CMV n=185

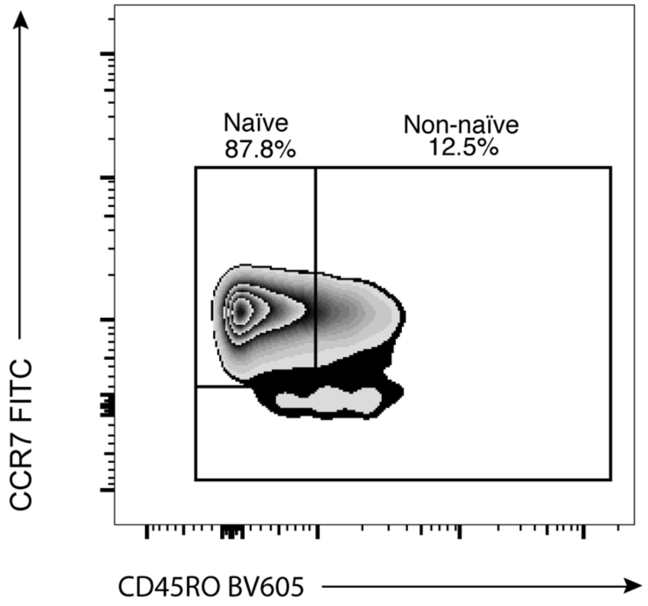

Supplemental Figure 1. Cord blood naïve and memory marker expression.
Representative flow plot of cord blood CD4 T cell naïve and non-naïve effector memory differentiation by CCR7 and CD45RO expression.

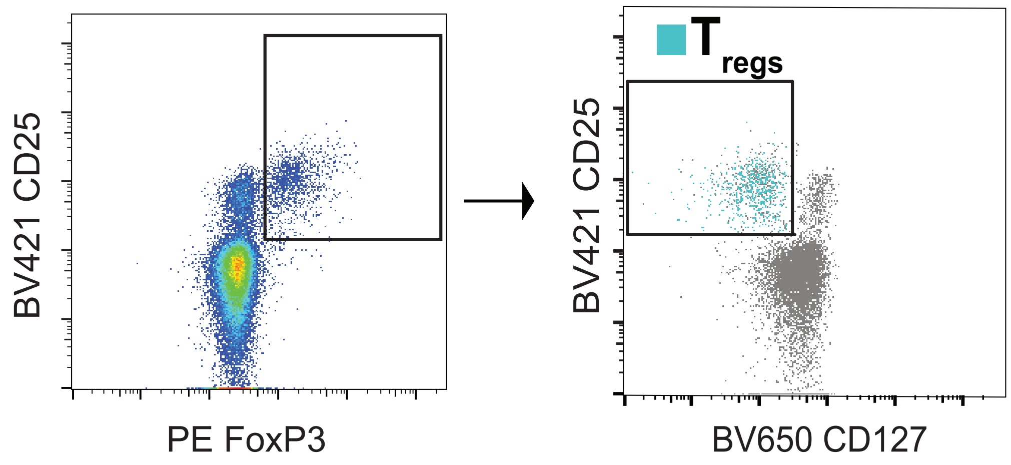

Supplemental Figure 2. Cord blood T regulatory cells.
Representative flow plot of cord blood CD4 T regulatory cells by CD25, FoxP3, and CD127 expression ($CD25^+FoxP3^+CD127^{lo}$). Cellular profiles were gated on live, single cell, dump negative (CD14, CD19, CD8), $CD3^+CD4^+$ lymphocytes.
