# Supplementary material for: Exposure to pesticides in utero impacts the fetal immune system and response to vaccination in infancy
Source: Nat Commun. 2021 Jan 8;12:132. doi: 10.1038/s41467-020-20475-8 (PMC7794579; doi:10.1038/s41467-020-20475-8)
Supplement: Supplementary file 3 — Reporting Summary [file 41467_2020_20475_MOESM3_ESM.pdf]

nature research

Corresponding author(s): Margaret Feeney, MD.

Last updated by author(s): Nov 20, 2020

Reporting Summary

Nature Research wishes to improve the reproducibility of the work that we publish. This form provides structure for consistency and transparency in reporting. For further information on Nature Research policies, seeAuthors & Referees and theEditorial Policy Checklist .

Statistics

For all statistical analyses, confirm that the following items are present in the figure legend, table legend, main text, or Methods section.

n/a	Confirmed	
☐	☒	The exact sample size (*n*) for each experimental group/condition, given as a discrete number and unit of measurement
☐	☒	A statement on whether measurements were taken from distinct samples or whether the same sample was measured repeatedly
☐	☒	The statistical test(s) used AND whether they are one- or two-sided<br>*Only common tests should be described solely by name; describe more complex techniques in the Methods section.*
☐	☒	A description of all covariates tested
☐	☒	A description of any assumptions or corrections, such as tests of normality and adjustment for multiple comparisons
☐	☒	A full description of the statistical parameters including central tendency (e.g. means) or other basic estimates (e.g. regression coefficient) AND variation (e.g. standard deviation) or associated estimates of uncertainty (e.g. confidence intervals)
☐	☒	For null hypothesis testing, the test statistic (e.g. *F*, *t*, *r*) with confidence intervals, effect sizes, degrees of freedom and *P* value noted<br>*Give P values as exact values whenever suitable.*
☒	☐	For Bayesian analysis, information on the choice of priors and Markov chain Monte Carlo settings
☒	☐	For hierarchical and complex designs, identification of the appropriate level for tests and full reporting of outcomes
☐	☒	Estimates of effect sizes (e.g. Cohen's *d*, Pearson's *r*), indicating how they were calculated

Our web collection on statistics for biologists contains articles on many of the points above.

Software and code

Policy information about availability of computer code

Data collection	STATA 14 (StataCorp), FlowJo software (Treestar), xPONENT software (v4.2)
Data analysis	PRISM 8.0 (GraphPad) and STATA 14 (StataCorp)

For manuscripts utilizing custom algorithms or software that are central to the research but not yet described in published literature, software must be made available to editors/reviewers. We strongly encourage code deposition in a community repository (e.g. GitHub). See the Nature Research guidelines for submitting code & software for further information.

Data

Policy information about availability of data

All manuscripts must include a data availability statement. This statement should provide the following information, where applicable:

- Accession codes, unique identifiers, or web links for publicly available datasets
- A list of figures that have associated raw data
- A description of any restrictions on data availability

The datasets generated during and/or analysed during the current study are available from the corresponding author on reasonable request

Field-specific reporting

Please select the one below that is the best fit for your research. If you are not sure, read the appropriate sections before making your selection.

☒ Life sciences ☐ Behavioural & social sciences ☐ Ecological, evolutionary & environmental sciences

For a reference copy of the document with all sections, see nature.com/documents/nr-reporting-summary-flat.pdf

Life sciences study design

All studies must disclose on these points even when the disclosure is negative.

Sample size	Samples were obtained from a malaria chemoprevention clinical trial of 300 HIV-negative pregnant women in Tororo, Uganda. 294 children were born between October 2014 and May 2015. Samples for immune parameters from cryopreserved samples (cord blood mononuclear cells, maternal peripheral blood mononuclear cells, or cord plasma) were chosen by 1) collection and successful banking of at least 2 vials of CBMCs or PBMCs, or at least 2 vials of cord plasma. 2) a recorded concentration of at least 8x10^6 CBMCs or PBMCs per vial or at least 1 mL of plasma. Variability in the number of of cells obtained post-thaw resulted in the inability to perform all immunological assays in all individuals. For whole blood absolute counts (dendritic cells and CD4 absolute counts), all participants with available whole cord blood at the time of delivery were sampled. For measles ELISA data, all children with at least one vial of plasma at 56 weeks of life was sampled. Samples taken for mass spectrometry evaluation of bendiocarb in maternal plasma were selected by 1) history of IRS exposure with bendiocarb during pregnancy 2) at least 1 vial of plasma available. Additionally we measured bendiocarb levels in the plasma 12 randomly selected women during pregnancy whose homes were not sprayed with IRS with bendiocarb prior to sample collection as a negative control. Using these sample sizes, we observed highly significant differences in evaluated immune parameters between bendiocarb exposed and unexposed participants, as well as dose-dependent changes in immune parameters based on plasma bendiocarb levels.
Data exclusions	No data was excluded. Subjects were excluded prior to performance of the assays if insufficient cells were available.
Replication	For flow cytometry data: To confirm reproducibility of assays and staining, a North American PBMC was included in phenotyping panels, for functional assays, a North American PBMC and North American CBMC control was included in all experiments. Due to limited number of available cells, all flow cytometry assays were performed on single replicates. For cord plasma cytokine and measles ELISA assays all samples were measured in duplicate and averaged between two replicates. Bendiocarb mass spectrometry assays were run in single replicates due to limited sample availability, but were validated on a Waters Ultra Performance Liquid Chromatography instrument. Bendiocarb analytic standard (Sigma Aldrich) was solubilized in methanol, and was validated over three separate intra inter day validation runs. Quality controls were run at low, medium and high levels over 6 replicates and the analysis was repeated over three days. Stability experiments were completed with 3 quality control levels at low and high concentrations.
Randomization	We compared participants with and without prenatal indoor residual spraying exposure with bendiocarb. Timing of IRS of each household was determined by the Government of Uganda without any influence from our study advisors.
Blinding	All clinical investigators who interacted with study subjects and recorded clinical outcomes were blinded to group assignment. Investigators who performed immunology assays were blinded to subjects' group assignment during data collection and analysis.

Reporting for specific materials, systems and methods

We require information from authors about some types of materials, experimental systems and methods used in many studies. Here, indicate whether each material, system or method listed is relevant to your study. If you are not sure if a list item applies to your research, read the appropriate section before selecting a response.

Materials & experimental systems

n/a	Involved in the study	
☐	☒	Antibodies
☒	☐	Eukaryotic cell lines
☒	☐	Palaeontology
☒	☐	Animals and other organisms
☐	☒	Human research participants
☐	☒	Clinical data

Methods

n/a	Involved in the study	
☒	☐	ChIP-seq
☐	☒	Flow cytometry
☒	☐	MRI-based neuroimaging

Antibodies

Antibodies used	CD3 APC/Cy7 (clone OKT3, lot# B247185, cat 317342, Biolegend, dilution 1:200); CD4 PerCP (clone RPA-T4, lot# B212868, cat 300528, Biolegend, dilution 1:100); CD25 BV421 (clone BC96, lot# B267415, cat 300528, Biolegend, dilution 1:200); CD127 BV650 (clone BC96, lot# B253988, cat 302630, Biolegend, dilution 1:200); CD45RO BV605 (clone A019D5, cat 351326, Biolegend, dilution 1:200); CCR7 FITC (clone UCHL1, lot# B194490, cat 353216, Biolegend, dilution 1:80); CD8 BV510 (clone SK1, lot# B230654, cat 344732, Biolegend, dilution 1:200); CD14 BV510 (clone M5E2, lot# B228386, cat 301842, Biolegend, dilution 1:200); CD19 BV510 (clone HIB19, lot# B23787, cat 302242, Biolegend, dilution 1:200); Ki67 FITC (clone Ki67, lot# 6077735, cat 51-365248, BD Pharmingen, dilution 1:200); FoxP3 PE (clone PCH101, lot#1955148, cat 12-4776-42, eBioscience, 1:200); Live/Dead aqua amine (lot# 2051092, cat L34965, Invitrogen, dilution 1:1666); CD3 PerCP (Clone SK7, lot# 3249560, cat 347344, BD Biosciences, 1:100); CD4 APC (clone RPA-T4, lot#B202682, cat 300514, BD Biosciences, dilution 1:250); Lin-2 FITC clone MϕP9/SJ25C1/SK7/L27/NCAM16.2, anti-CD3/CD14/CD19/CD20/CD56, lot# 4002915, cat 643397, BD Bioscience, dilution 1:250); CD123 PE (Clone 9F5, lot# 3256989, cat 340545, BD Bioscience, dilution 1:250); CD11c APC (clone S-HCL-3, lot#6200808, cat 340544, BD Bioscience, dilution 1:250). CD3 PE-Cy5.5 (clone SK7, lot #4306773, eBioscience, dilution 1:150); CD4 BV570 (clone RPA-T4, lot #B222263, Biolegend dilution 1:100); CD8 BV711 (clone RPA-T8, lot #B227046, Biolegend, dilution 1:200); CD45RA BV650 (clone HI100, lot #B210772, Biolegend, dilution 1:100); CD45RO BV605 (clone UCHL1, lot #B213399, Biolegend, dilution 1:100); gdTCR BV510 (clone B1, lot #B210008, Biolegend, dilution 1:200); CD14 BV510 (clone M5E2, lot #B228386, Biolegend dilution

	1:200); CD19 BV510 (clone HIB19, lot #B198603, Biolegend, dilution 1:200); TNF-alpha PE-e610 (clone MAB11, lot#E24914-101, eBioscience, dilution 1:50); IFN-gamma PE-Cy7 (clone 4S.B.3, lot #B193275, dilution 1:100, BD); IL-2 BV421 (clone MQ1-17H12, lot #B205616, Biolegend, dilution 1:100); IL-8 AF488 (clone E8N1, lot #B161916, Biolegend, dilution 1:200); CFSE (Molecular probes, dilution 3-4 x 10^6 cells were labeled with 1 µM CFSE)
Validation	Validation and titration of all antibodies was performed on healthy human North American peripheral blood mononuclear cells with fluorescence-minus-one samples to define negative and positive population.

Human research participants

Policy information about studies involving human research participants

Population characteristics	The parent clinical trial of maternal chemoprevention enrolled 300 HIV-negative pregnant women were enrolled between June 2014-October 2014 in Tororo, Uganda. 300 HIV-negative pregnant women were enrolled between 12-20 weeks gestation and randomized to receive either dihydroartemisinin-piperaquine (DP) or sulfadoxine-pymethamine (SP) for intermittent preventive treatment of malaria (ClinicalTrials.gov number, NCT02163447).
Recruitment	The presented work uses biobanked specimens collected from a parent study of a clinical trial of malaria chemoprevention. The parent clinical trial screened 386 pregnant women in 2014, and enrolled 300 women. Complete recruitment details are available in a prior publication (Kakuru, A. et al. Dihydroartemisinin-Piperaquine for the Prevention of Malaria in Pregnancy. NEJM 2016.). To limit selection bias of samples, sample selection criteria was determined prior to sample selection. Samples for immune parameters from cryopreserved samples (cord blood mononuclear cells, maternal peripheral blood mononuclear cells, or cord plasma) were chosen by 1) collection and successful banking of at least 2 vials of CBMCs or PBMCs, or at least 2 vials of cord plasma. 2) a recorded concentration of at least 8x10^6 CBMCs or PBMCs per vial or at least 1 mL of plasma. Variability in the number of of cells obtained post-thaw resulted in the inability to perform all immunological assays in all individuals. For whole blood absolute counts (dendritic cells and CD4 absolute counts), all participants with available whole cord blood at the time of delivery were sampled. For measles ELISA data, all children with at least one vial of plasma at 56 weeks of life was sampled. Samples taken for mass spectrometry evaluation of bendiocarb in maternal plasma were selected by 1) history of IRS exposure with bendiocarb during pregnancy 2) at least 1 vial of plasma available. Additionally we measured bendiocarb levels in the plasma 12 randomly selected women during pregnancy whose homes were not sprayed with IRS with bendiocarb prior to sample collection as a negative control
Ethics oversight	Institutional review boards of the Uganda National Council of Science and Technology (UNCST), Makerere University, and the University of California San Francisco (UCSF) approved the study. Informed consent was obtained from all study participants.

Note that full information on the approval of the study protocol must also be provided in the manuscript.

Clinical data

Policy information about clinical studies

All manuscripts should comply with the ICMJEguidelines for publication of clinical research and a completedCONSORT checklist must be included with all submissions.

Clinical trial registration	The presented work uses biobanked specimens collected from a parent study of a clinical trial of malaria chemoprevention (ClinicalTrials.gov number, NCT02163447).
Study protocol	The presented work uses biobanked specimens from collected from a parent study of a clinical trial of malaria chemoprevention (ClinicalTrials.gov number, NCT02163447). The parent study protocol is available at https://www.nejm.org/doi/suppl/10.1056/NEJMoa1509150/suppl_file/nejmoa1509150_protocol.pdf
Data collection	The presented work uses biobanked specimens from collected from a parent study of a clinical trial of malaria chemoprevention (ClinicalTrials.gov number, NCT02163447). The parent study protocol and data collections methods is available at https://www.nejm.org/doi/suppl/10.1056/NEJMoa1509150/suppl_file/nejmoa1509150_protocol.pdf
Outcomes	The presented work uses biobanked specimens from collected from a parent study of a clinical trial of malaria chemoprevention (ClinicalTrials.gov number, NCT02163447). The parent study protocol and outcomes is available at https://www.nejm.org/doi/suppl/10.1056/NEJMoa1509150/suppl_file/nejmoa1509150_protocol.pdf

Flow Cytometry

Plots

Confirm that:

- [x] The axis labels state the marker and fluorochrome used (e.g. CD4-FITC).
- [x] The axis scales are clearly visible. Include numbers along axes only for bottom left plot of group (a 'group' is an analysis of identical markers).
- [x] All plots are contour plots with outliers or pseudocolor plots.
- [x] A numerical value for number of cells or percentage (with statistics) is provided.

Methodology

Sample preparation	For cryopreserved samples, frozen CBMCs or PBMCs were thawed, washed twice with complete media (RPMI + 10% FBS +Penicillin Streptomycin + HEPES + L-glutamine + DNase). Cell counts were performed using a Muse cell analyzer and CBMCs were resuspended in PBS for flow cytometry staining or complete media for ICS or CFSE dilution experiments. For whole blood flow cytometry analysis, whole blood was added to antibody stain and incubated. BD FACS lysis buffer was then added and incubated. CountBright counting beads (ThermoFisher Scientific) were added and samples were analyzed on a flow cytometer.
Instrument	Data for cryopreserved samples were collected on an LSR II (BD), and for whole blood samples Accuri A6 cytometer
Software	Data was analyzed with FlowJo Software (TreeStar)
Cell population abundance	1x10^6 CBMCs or PBMCs was used per flow cytometry condition
Gating strategy	Surface and intracellular staining of thawed CBMCs and PBMCs was performed with standard protocols, using antibodies as listed in methods. All samples were stained with LIVE/DEAD aqua amine (Invitrogen) to discriminate live from dead cells and a "dump' channel with CD14 and CD19 for exclusion gating. The gating strategy for CD4 naive and non-naive populations is included in Supp. Fig. 1. The gating strategey for Tregs is included in Supp Fig. 2. The gating strategy for CD4 and CD8 ICS and CFSE dilution assays are included in Fig 3.

☒ Tick this box to confirm that a figure exemplifying the gating strategy is provided in the Supplementary Information.
