# Supplementary material for: Exposure to pesticides in utero impacts the fetal immune system and response to vaccination in infancy
Source: Nat Commun. 2021 Jan 8;12:132. doi: 10.1038/s41467-020-20475-8 (PMC7794579; doi:10.1038/s41467-020-20475-8)
Supplement: Supplementary file 2 — Peer Review File [file 41467_2020_20475_MOESM2_ESM.pdf]

Editorial Note: This manuscript has been previously reviewed at another journal that is not operating a transparent peer review scheme. The manuscript was considered suitable for publication without further review at Nature Communications.
